# Supplementary material for: Simple, Low-Cost and Long-Lasting Film for Virus Inactivation Using Avian Coronavirus Model as Challenge
Source: Int J Environ Res Public Health. 2020 Sep 4;17(18):6456. doi: 10.3390/ijerph17186456 (PMC7558860; doi:10.3390/ijerph17186456)
Supplement: Supplementary file 1 [file ijerph-17-06456-s001.pdf]

Table S1. The p values from comparisons between experimental groups (film + challenge and respective positive control group) for Log10 ACoV RNA copies and qualitative results (virus isolation and RT-qPCR).

	**Parameter**	**Hands**	**Inanimate surface**
**High challenge**	RT-qPCR (quantitative)	0.0041	0.0028
	Virus isolation	0.0152	0.0022
	RT-qPCR (qualitative)	0.0022	0.0022
**Intermediate challenge**	RT-qPCR (quantitative)	0.0028	0.0021
	Virus isolation	0.0022	0.0152
	RT-qPCR (qualitative)	0.0022	0.0022
**Low challenge**	RT-qPCR (quantitative)	0.0021	0.0015
	Virus isolation	0.0152	0.0013
	RT-qPCR (qualitative)	0.0022	0.0025
